# Cooperative modular multilevel converter control based on PSO-optimized fuzzy-PI and hierarchical finite-state model predictive control

**DOI:** 10.1038/s41598-025-30005-5

**Published:** 2025-11-24

**Authors:** Yale Liu, Yizhi Tian, Wenjie Zhang, Yixuan Wang

**Affiliations:** School of Electrical engineering, Xinjiang University, Urumqi, China

**Keywords:** Modular multilevel converter, Particle swarm optimization, Fuzzy PI control, Model predictive control, Engineering, Mathematics and computing

## Abstract

To address the limitations of empirical parameter tuning in the outer-loop PI controller and the high computational burden of the inner loop in conventional dual-loop control for modular multilevel converters, a coordinated control strategy is proposed that integrates particle swarm optimization-based fuzzy PI control with finite-state hierarchical model predictive control. The outer loop employs a two-dimensional fuzzy PI controller, in which error and error variation are selected as inputs. The quantization and proportional factors are optimally determined through an offline global search using particle swarm optimization, enabling adaptive parameter adjustment and improving the tracking accuracy of current reference signals under varying operating conditions. The inner loop adopts finite-state model predictive control, where AC-side phase voltages and total arm voltages are predicted using a reverse calculation method. Based on objective function minimization combined with integer rounding, the required number of submodules for the upper and lower arms is determined. Circulating current suppression is subsequently introduced to mitigate steady-state deviations on the AC side, and predictive submodule voltage grouping and sorting are applied to balance capacitor voltages. This approach eliminates the need for weighting factor design and effectively reduces computational complexity. Finally, simulations carried out in MATLAB/Simulink confirm the effectiveness of the proposed strategy.

## Introduction

With increasing penetration of renewable energy and the growing demand for flexible grid regulation, the modular multilevel converter (MMC) has become a key topology for high-voltage direct-current (HVDC) transmission^1,2^ and large-capacity power conversion owing to its modular architecture, scalability^3,4^, and high-quality output waveforms^5,6^. MMC control typically adopts a dual-loop framework: the outer loop generates current references according to system energy management and station-level scheduling, while the inner loop performs current tracking together with circulating-current suppression and submodule capacitor-voltage balancing^7–9^. The coordinated design of these loops determines both dynamic performance and practical feasibility. Therefore, an effective control strategy should first enhance the accuracy of outer-loop reference generation and, on that basis, reduce the computational burden of the inner loop to improve the real-time performance of the overall control system.

In the outer loop, conventional proportional–integral (PI) controllers are widely used due to their simplicity and industrial maturity. However, fixed PI gains imply a fundamental trade-off between transient speed and overshoot attenuation, and controller performance is sensitive to parameter drift and operating-point variations^10–12^. To increase robustness, fuzzy logic has been integrated into PI architectures, enabling adaptive gain adjustment based on error and error-rate information and thereby improving transient and steady-state responses^13–15^. Nevertheless, fuzzy PI schemes require selection of quantization and scaling factors, which are typically tuned manually; such manual tuning is time-consuming and rarely guarantees globally effective parameter sets across diverse operating scenarios^16^. This motivates the adoption of systematic optimization methods that retain fuzzy adaptation while achieving globally tuned parameters for more accurate outer-loop reference generation.

In the inner loop, finite control set model predictive control (FCS-MPC) has been widely applied to MMCs thanks to its ability to handle multivariable and nonlinear dynamics while offering fast transient response^17–19^. Its cost function usually includes current tracking, circulating current suppression, and capacitor voltage balancing^20,21^. Nevertheless, conventional FCS-MPC requires evaluating a large number of switching states at each sampling instant, leading to an exponential increase in computational complexity with more submodules. In addition, the weighting factors of multi-objective cost functions are typically selected empirically, which complicates the balance among competing objectives. To mitigate these drawbacks, researchers have proposed improved MPC strategies, such as submodule voltage sorting to reduce switching evaluations^22^ or restricting capacitor voltage ranges to limit the search space under faults^23^. Although these methods relieve computational stress, they still face issues of weight selection and scalability. Recent works have proposed computationally efficient MPC variants and reduced finite-set or preprocessing strategies to enable real-time implementation^24,25^. Hierarchical strategies^26,27^ have been introduced to optimize objectives sequentially and avoid weight tuning, but their computational burden still scales with the number of submodules, and the AC side remains constrained to *N* + 1 voltage levels.

To address the above challenges, this paper proposes a coordinated dual-loop control strategy that integrates a particle-swarm-optimization (PSO)-tuned fuzzy-PI outer loop with a hierarchical FCS-MPC inner loop. PSO is applied offline to determine fuzzy quantization and proportional–integral parameters using the integral of time-weighted absolute error (ITAE) as the optimization objective, thus improving outer-loop reference quality. Instantaneous-power theory and inverse Park transformation are used to convert optimized power commands into three-phase current references for the inner loop. The inner loop, implemented on a discrete MMC model, combines candidate-set constraints, predictive voltage grouping, and an error-compensation mechanism in circulating-current suppression to substantially reduce switching-state evaluations and effective switching frequency while maintaining accurate current tracking and capacitor-voltage balancing. MATLAB/Simulink simulations demonstrate that the proposed method yields faster transients, lower steady-state error, and reduced computational overhead compared with conventional dual-loop schemes.

## MMC submodule topology and modeling basics

**Fig. 1:**
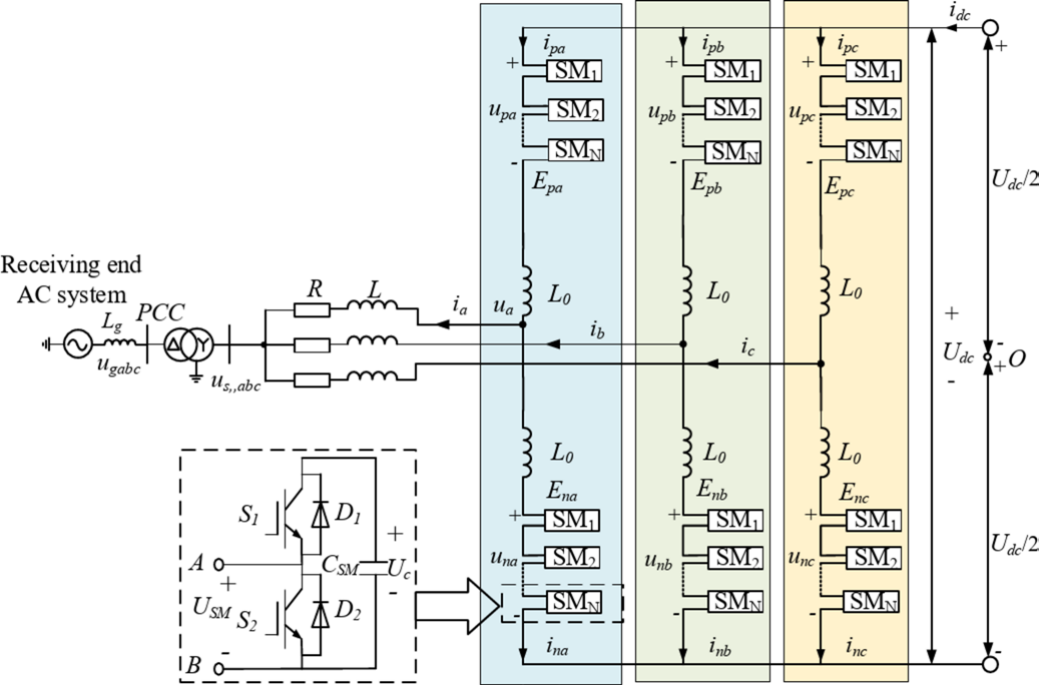
MMC main circuit topology diagram.

The main circuit topology of the MMC is shown in Fig. 1. The structure is three-phase symmetric, with each phase composed of an upper and a lower bridge arm. Each bridge arm contains *N* series-connected half-bridge submodules, which consist of power switches, anti-parallel diodes, and energy-storage capacitors. These submodules are the key units for voltage synthesis and energy exchange.

The switching state of a submodule is determined by the on/off operation of its IGBTs. When the upper IGBT is on and the lower IGBT is off, the submodule is inserted into the circuit, the capacitor is connected to the bridge arm, and the switching state is defined as *S* = 1. Conversely, when the lower IGBT is on and the upper IGBT is off, the submodule is bypassed, the capacitor is short-circuited, and the switching state is defined as *S* = 0.

Each bridge arm is connected in series with an arm inductor *L*_0_, whose main function is to suppress sudden current variations that could damage the IGBTs and to mitigate the rate of circulating current changes.

To enable precise outer-loop power regulation and coordinated inner-loop multi-objective control, it is essential to establish the mathematical model of the MMC system and to characterize the dynamic relationships among key variables, including the DC-link voltage, arm voltages, AC currents, circulating currents, and submodule capacitor voltages. This modeling forms the foundation for the subsequent controller design.

Given the inherent symmetry of the three-phase structure, only the j-phase(*j* = a, b,c) isconsidered here for derivation. On the AC side, the relevant variables are the grid voltage *u*_*s, j*_ and the output current *i*_*j*_.The arm variables include the upper and lower arm voltages (*u*_*pj*_ and *u*_*nj*_) as well as their corresponding currents(*i*_*p, j*_ and *i*_*n, j*_). On the DC side, the key quantities are the DC-link voltage Udc and the DC current *I*_*dc*_.

Each arm is composed of n cascaded half-bridge submodules. The arm voltage is determined jointly by the capacitor voltages of the individual submodules and their switching states. Taking the *j*-phase as an example, the arm voltage can be expressed as:

1 $${u_{p,j}} = \mathop \sum \limits_{k = 1}^n {S_{p,j,k}} \cdot {u_{sm,p,j,k}}$$

2 $${u_{n,j}} = \mathop \sum \limits_{k = 1}^n {S_{n,j,k}} \cdot {u_{sm,n,j,k}}$$

where *u*_*p, j*_ and *u*_*n, j*_ denote the total output voltages of the upper and lower arms of phase *j*, obtained by summing the capacitor voltages of all inserted submodules in each arm; *S*_*p, j,k*_ and *S*_*n, j,k*_ denote the switching states (*S*∈{0,1}) of the *k*-th submodule in the upper and lower arms of phase *j*, respectively, and *u*_*sm, p,j, k*_ and *u*_*sm, n,j*_, represent the corresponding capacitor voltages.

For the two loops formed by the upper and lower arms together with the DC link, Kirchhoff’s voltage law (KVL) yields:

3 $$\frac{{{U_{dc}}(t)}}{2}-{u_{pj}}(t)-{L_0}\frac{{d{i_{pj}}(t)}}{{dt}}=R{i_j}(t)+L\frac{{d{i_j}(t)}}{{dt}}$$

4 $$\frac{{{U_{dc}}(t)}}{2} - {u_{nj}}(t) - {L_0}\frac{{d{i_{nj}}(t)}}{{dt}}= - R{i_j}(t) - L\frac{{d{i_j}(t)}}{{dt}}$$

where *U*_*dc*_ denotes the DC-link voltage of the converter; *R* is the arm resistance (including the on-state resistance of the submodule IGBTs), *L*_0_ is the arm inductance and *L* is the AC-side filter inductance; while *i*_*p, j*_, *i*_*n, j*_, and *i*_*j*_ correspond to the upper-arm, lower-arm, and AC-side currents.

To characterize the dynamic behavior of the AC current and circulating current, the *j*-phase AC current and the arm internal current are defined as:

5 $${i_j}={i_{p,j}} - {i_{n,j}}$$

6 $${i_{diff,j}}=\frac{{{i_{p,j}}+{i_{n,j}}}}{2}={i_{c,j}}+\frac{{{i_{dc}}}}{3}$$

where *i*_*diff, j*_ is the internal current of the bridge arm, which is determined by the direct current component *i*_*dc*_/3 and the loop current *i*_*c, j*_.

By substituting Eqs. (5)-(6) into Eqs. (3)-(4) and eliminating *i*_*p, j*_ and *i*_*n, j*_, the dynamic equations of the AC current and the internal arm current are obtained, as summarized in Eqs. (7)-(8):

7 $$\left( {\begin{array}{*{20}{c}} {L+\frac{{{L_0}}}{2}} \end{array}} \right)\frac{{d{i_j}}}{{dt}}+R{i_j}=\frac{{{u_{p,j}} - {u_{n,j}}}}{2} - {u_{s,j}}$$

8 $${L_0}\frac{{d{i_{diff,j}}}}{{dt}}=\frac{{{U_{dc}}}}{2} - \frac{{{u_{p,j}}+{u_{n,j}}}}{2}$$

Equation (7) gives the three-phase dynamic model of the MMC in the stationary reference frame. In steady-state operation, the MMC output is sinusoidal, which is not convenient for controller design. To enable a more straightforward and stable control strategy, a *dq* transformation is applied to convert the three-phase sinusoidal variables in the stationary frame into two DC quantities in the synchronous rotating frame. The transformation matrix Γ(θ) is expressed as:

9 $${\mathrm{\boldsymbol{\Gamma}(\boldsymbol{\uptheta})=}}\frac{{\mathrm{2}}}{{\mathrm{3}}}\left[ \begin{gathered} \begin{array}{*{20}{c}} {{\mathrm{cos\boldsymbol{\uptheta}}}}&{{\mathrm{cos(\boldsymbol{\uptheta}-}}\frac{{{\mathrm{2\boldsymbol{\uppi}}}}}{{\mathrm{3}}}{\mathrm{)}}}&{{\mathrm{cos(\boldsymbol{\uptheta}+}}\frac{{{\mathrm{2\boldsymbol{\uppi}}}}}{{\mathrm{3}}}{\mathrm{)}}} \end{array} \hfill \\ \begin{array}{*{20}{c}} {{\mathrm{sin\boldsymbol{\uptheta}}}}&{{\mathrm{sin(\boldsymbol{\uptheta}-}}\frac{{{\mathrm{2\boldsymbol{\uppi}}}}}{{\mathrm{3}}}{\mathrm{)}}}&{{\mathrm{sin(\boldsymbol{\uptheta}-}}\frac{{{\mathrm{2\boldsymbol{\uppi}}}}}{{\mathrm{3}}}{\mathrm{)}}} \end{array} \hfill \\ \begin{array}{*{20}{c}} {\frac{{\mathrm{1}}}{{\mathrm{2}}}\begin{array}{*{20}{c}} {}&{\begin{array}{*{20}{c}} {}&{} \end{array}} \end{array}}&{\frac{{\mathrm{1}}}{{\mathrm{2}}}\begin{array}{*{20}{c}} {}&{}&{} \end{array}}&{\frac{{\mathrm{1}}}{{\mathrm{2}}}} \end{array} \hfill \\ \end{gathered} \right]$$

where *θ* denotes the phase angle of the phase-a voltage of the AC system. By substituting Eq. (9) into Eq. (7), the following *dq*-axis dynamic model is obtained, as shown in Eq. (10):

10 $$L\frac{d}{{dt}}\left[ {\begin{array}{*{20}{c}} {{i_d}} \\ {{i_q}} \end{array}} \right]+R\left[ {\begin{array}{*{20}{c}} {{i_d}} \\ {{i_q}} \end{array}} \right]=\left[ {\begin{array}{*{20}{c}} {{u_d}} \\ {{u_q}} \end{array}} \right] - \left[ {\begin{array}{*{20}{c}} {{v_d}} \\ {{v_q}} \end{array}} \right]+\left[ {\begin{array}{*{20}{c}} {{\boldsymbol{\omega}_L}} \\ { - {\boldsymbol{\omega}_L}} \end{array}} \right]\left[ {\begin{array}{*{20}{c}} {{i_d}} \\ {{i_q}} \end{array}} \right]$$

where *i*_*d*_ and *i*_*q*_ represent the *d*- and *q*-axis components of the AC-side current, respectively; *u*_*d*_ and *u*_*q*_ are the corresponding *d*- and *q*-axis components of the grid voltage; and *ω* is the angular frequency of the AC system.

According to the instantaneous power theory, the active and reactive power of the AC system can be expressed as:

11 $$\left[ {\begin{array}{*{20}{c}} P \\ Q \end{array}} \right]=\frac{3}{2}\left[ {\begin{array}{*{20}{c}} {{u_{sd}}}&{{u_{sq}}} \\ {{u_{sq}}}&{ - {u_{sd}}} \end{array}} \right]\left[ {\begin{array}{*{20}{c}} {{i_d}} \\ {{i_q}} \end{array}} \right]$$

The phase angle *θ* of the phase-a voltage is measured using a phase-locked loop (PLL). Together with the *abc/dq* coordinate transformation, the three-phase voltages are converted into the synchronous rotating reference frame. Under balanced three-phase conditions, the *q*-axis component *u*_*sq*_ of the AC-side voltage equals zero, and Eq. (11) for the instantaneous active and reactive power can be simplified as shown in Eq. (12):

12 $$\begin{array}{*{20}{c}} {P=\frac{3}{2}{u_{sd}}{i_d}} \\ {Q= - \frac{3}{2}{u_{sd}}{i_q}} \end{array}$$

where *u*_*sd*_ is the *d*-axis component of the AC-side voltage.

From Eq. (12), the active power depends solely on *i*_*d*_ ,while the reactive power depends solely on *i*_*q*_, enabling their independent regulation. The reference three-phase currents *i*^∗^_*abc*_ are then obtained through the inverse T_*dq/abc*_ transformation.

## Dual-loop cooperative control strategy based on PSO-fuzzy PI and hierarchical finite-state MPC

**Fig. 2:**
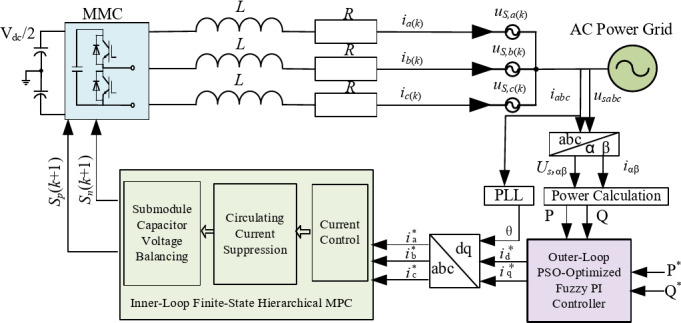
Block diagram of optimal control strategy for MMC inverter.

To achieve fast response and high-precision power tracking, the proposed strategy retains the conventional dual-loop framework, but improves both loops: the outer loop adopts a PSO-based fuzzy PI controller for accurate current reference generation, while the inner loop employs hierarchical FCS-MPC to decouple multi-objective control and reduce computational complexity. The overall framework is shown in Fig. 2.

### Outer ring PSO fuzzy PI controller design

The outer loop generates accurate *dq*-axis current references from power commands, serving as a key factor in both dynamic response and steady-state accuracy. Conventional PI controllers with fixed parameters often suffer from delayed responses, excessive overshoot, and difficulty suppressing steady-state errors under disturbances or changing operating conditions. To enhance adaptability, a fuzzy PI controller is adopted, where the proportional and integral gains *K*_*p*_ and integral gain *K*_*i*_ are dynamically adjusted in real time through fuzzy inference rules.

In this design, the controller takes the power tracking error *e* and its rate of change *ec* as inputs. After scaling by quantization factors *α*_*e*_ and *α*_*ec*_, they are normalized to the range [− 3,3]. The outputs, Δ*K*_*p*_ and Δ*K*_*i*_, are constrained within [− 1,1]. Both input and output variables employ triangular membership functions divided into seven subsets.

**Fig. 3:**
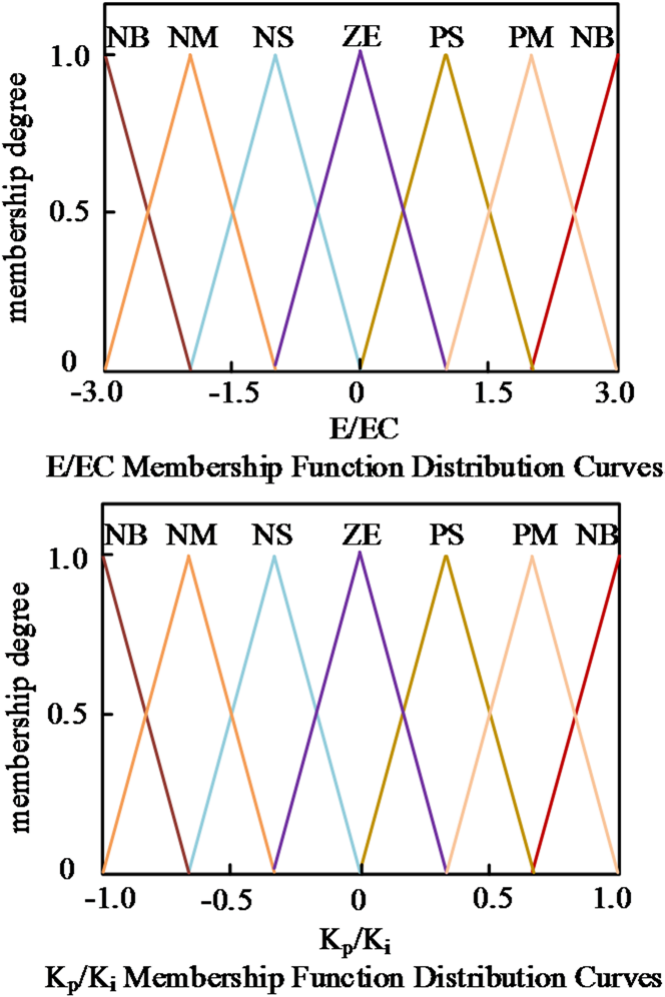
Membership functions of inputs and outputs.

{*NB*,*NM*,*NS*,*ZE*,*PS*,*PM*,*PB*}, representing negative large, negative medium, negative small, zero, positive small, positive medium, and positive large. The membership functions and their centers are illustrated in Fig. 3.

To facilitate quantitative computation in the fuzzy inference process, the linguistic variables of the input quantities (E and EC) are numerically mapped to corresponding membership grades, as summarized in Table 1. This table defines the assignment relationship between linguistic labels and their numeric levels, providing a clear basis for fuzzification and subsequent rule-base construction.

**Table 1:** Linguistic-to-numeric assignment table for E and EC fuzzy variables.

e(ec) E(EC)	-3	-2	-1	0	1	2	3
PB	0	0	0	0	0.5	1	1
PM	0	0	0	0.5	1	0.5	0
PS	0	0	0.5	1	0.5	0	0
ZO	0	0.5	1	0.5	0	0	0
NS	0	1	0.5	0	0	0	0
NM	0.5	1	0.5	0	0	0	0
NB	1	0.5	0	0	0	0	0

To ensure satisfactory system performance, the regulation rules of *ΔK*_*p*_ and *ΔK*_*i*_ were derived from PI tuning characteristics and expert knowledge:

- When the tracking error *E* is large, increase *K*_*p*_ and decrease *K*_*i*_ to accelerate the dynamic response and quickly reduce the error.
- As the error decreases, both *K*_*p*_ and *K*_*i*_ should be reduced to suppress overshoot and maintain stable operation.
- When the error approaches zero, the system nears steady state; in this case, further decrease *K*_*p*_ and appropriately increase *K*_*i*_ to eliminate residual steady-state error.
- If *E* and *EC* share the same sign, the system is diverging from the target, and *K*_*i*_ should be reduced to promote convergence. If they have opposite signs, the system is converging toward the target, and *K*_*p*_ should be reduced to improve dynamic smoothness.

Based on these rules, a 7 × 7 fuzzy rule table for Δ*K*_*p*_ and Δ*K*_*i*_ is established, as shown in Table 2.

**Table 2:** Fuzzy control rules for ΔKp/ΔKi controls.

EC	Control rules						
	E = NB	E = NM	E = NS	E = ZE	E = PS	E = PM	E = PB
NB	PB/NB	PB/NB	PM/NM	PM/NM	PS/NS	ZE/ZE	ZE/ZE
NM	PB/NB	PB/NB	PM/NM	PS/NS	PS/NS	ZE/ZE	NS/ZE
NS	PM/NB	PM/NM	PM/NS	PS/NS	ZE/ZE	NS/PS	NS/PS
ZE	PM/NM	PM/NM	PS/NS	ZE/ZE	NS/PS	NM/PM	NM/PM
PS	PS/NM	PS/NS	ZE/ZE	NS/PS	NS/PS	NM/PM	NM/PB
PM	PS/ZE	ZE/ZE	NS/PS	NM/PS	NM/PM	NM/PB	NB/PB
PB	ZE/ZE	ZE/ZE	NM/PS	NM/PM	NM/PM	NB/PB	NB/PB

Mamdani-type fuzzy inference was adopted, with min–max operations used for rule evaluation and aggregation, and defuzzification performed by the centroid method. Let *A*_*i*_,*B*_*j*_∈{*NB*,*NM*,*NS*,*ZE*,*PS*,*PM*,*PB*} denote the linguistic terms of *E* and *EC*, and *C*_*p, ij*_,*C*_*i, ij*_ the outputs. The fuzzy rule *R*_*ij*_ is expressed as:

R_ij_: IF *E* is *A*_*i*_ AND *EC* is *B*_*j*_ THEN *ΔK*_*p*_ is *C*_*p, ij*_, *ΔK*_*i*_ is *C*_*i, ij*_.

Trigger strength:

13 $${\omega _{ij}}={\mathrm{min}}\left( {\begin{array}{*{20}{c}} {{\mu _{{A_i}}}(E),{\mu _{{B_j}}}(EC)} \end{array}} \right)$$

Aggregate output:

14 $${\mu _{\Delta {K_p}}}(x) = \mathop {{\mathrm{max}}}\limits_{{\mathrm{i,j}}} {\mathrm{min}}\left( {{\omega _{ij}},{\mu _{{C_{p,ij}}}}(x)} \right)$$

15 $${\mu _{\Delta {K_i}}}(x) = \mathop {{\mathrm{max}}}\limits_{{\mathrm{i,j}}} {\mathrm{min}}\left( {{\omega _{ij}},{\mu _{{C_{i,ij}}}}(x)} \right)$$

The centroid method was applied to obtain the crisp outputs Δ*K*_*p*_ and Δ*K*_*i*_:

16 $$\Delta {K_p} = \frac{{\mathop \sum \limits_{k = 1}^N {x_k}{\mu _{\Delta {K_p}}}({x_k})}}{{\mathop \sum \limits_{k = 1}^N {\mu _{\Delta {K_p}}}({x_k})}}$$

17 $$\Delta {K_i} = \frac{{\mathop \sum \limits_{k = 1}^N {x_k}{\mu _{\Delta {K_i}}}({x_k})}}{{\mathop \sum \limits_{k = 1}^N {\mu _{\Delta {K_i}}}({x_k})}}$$

where *ω*_*ij*_ denotes the firing strength of the (*i*,*j*)-th fuzzy rule; *N* is the total number of sampling points, *x*_*k*_ denotes the discrete points in the output domain, and *µ*(*x*_*k*_) represents the membership function value of the corresponding output.

The defuzzified results were scaled by the output factors *β*_*Kp*_ ,*β*_*Ki*_ and superimposed on the initial values (with saturation protection) to yield the final controller gains:

18 $${K_p}=\mathrm{s}\mathrm{a}\mathrm{t}\left( {{K_{p0}}+{\beta _{Kp}}\Delta {K_p}} \right)$$

19 $${K_i}=\mathrm{s}\mathrm{a}\mathrm{t}\left( {{K_{i0}}+{\beta _{Ki}}\Delta {K_i}} \right)$$

where sat denotes the limiting and anti-saturation processing, and *K*_*p0*_ and *K*_*i0*_ are the initial values of the gain.

**Fig. 4:**
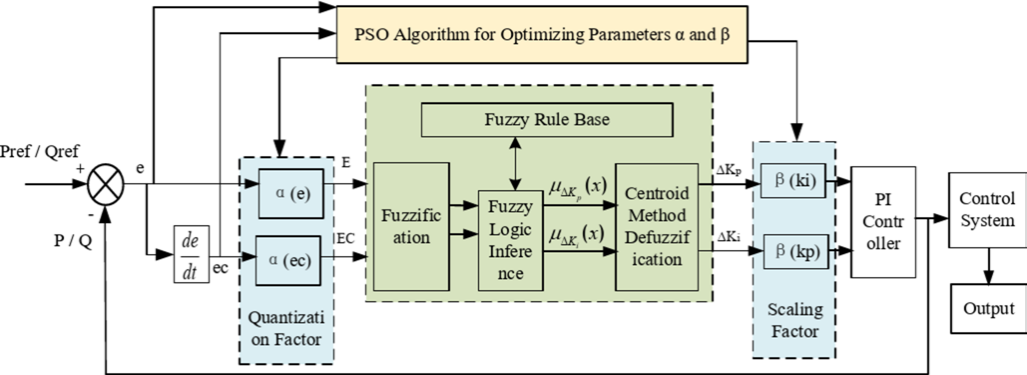
Particle swarm optimised fuzzy PI control structure.

The quantization factors (*α*_*e*_,*α*_*ec*_) determine the sensitivity of input mapping, while the scaling factors (*β*_*Kp*_, *β*_*Ki*_) define the magnitude of output adjustment. Both have a critical impact on control performance. However, manual tuning of these parameters often fails to achieve global optimality across varying operating conditions. To address this, particle swarm optimization (PSO), a population-based intelligent search algorithm, was employed to offline optimize [*α*_*e*_, *α*_*ec*_, *β*_*Kp*_, *β*_*Ki*_]. By leveraging iterative information exchange and cooperative search among particles, PSO enhances the robustness and adaptability of the fuzzy PI controller. The structure of the PSO-optimized fuzzy PI controller is illustrated in Fig. 4.

The PSO algorithm initializes a swarm of particles, each representing a candidate solution for the fuzzy PI controller parameters and characterized by position and velocity. The particle position is defined as a four-dimensional vector: X_i_=[*α*_*e*_, *α*_*ec*_, *β*_*Kp*_, *β*_*Ki*_], During each iteration, the algorithm updates particle velocity and position by combining individual and global optima to approach the best solution:

20 $${v_{id}}(k+1)=w{v_{id}}(k)+{c_1}{r_1}({p_{id}}(k) - {x_{id}}(k))+{c_2}{r_2}({p_{gd}}(k) - {x_{id}}(k))\;\;\:$$

21 $${x_{id}}(k+1)={x_{id}}(k)+{v_{id}}(k+1)$$

where *v*_*id*_(*k*) and *x*_*id*_(*k*) are the velocity and position of the *i*-th particle in the *d*-th dimension at iteration *k*; *p*_*id*_(*k*) and *p*_*gd*_(*k*) are the personal and global best positions; *c*_1_ and *c*_2_ are learning coefficients; and *r*_1_,*r*_2_ are random numbers.

The inertia weight *w* is linearly decreased to balance global exploration and convergence speed:

22 $$w(t)=\frac{{{w_{start}} - \left( {{w_{start}} - {w_{end}}} \right)t}}{{{t_{\hbox{max} }}}}$$

where *t* and *t*_max_ denote the current and maximum iterations, and *w*_start_, *w*_end_ are the initial and final weights.

The fitness of each particle is evaluated using the Integral of Time-weighted Absolute Error (ITAE):

23 $$ITAE = \smallint _0^\infty t|e(t)|dt$$

where *e*(*t*) represents the error between the system output and the reference. ITAE comprehensively reflects both transient speed and steady-state accuracy, thus improving the controller’s performance.

**Fig. 5:**
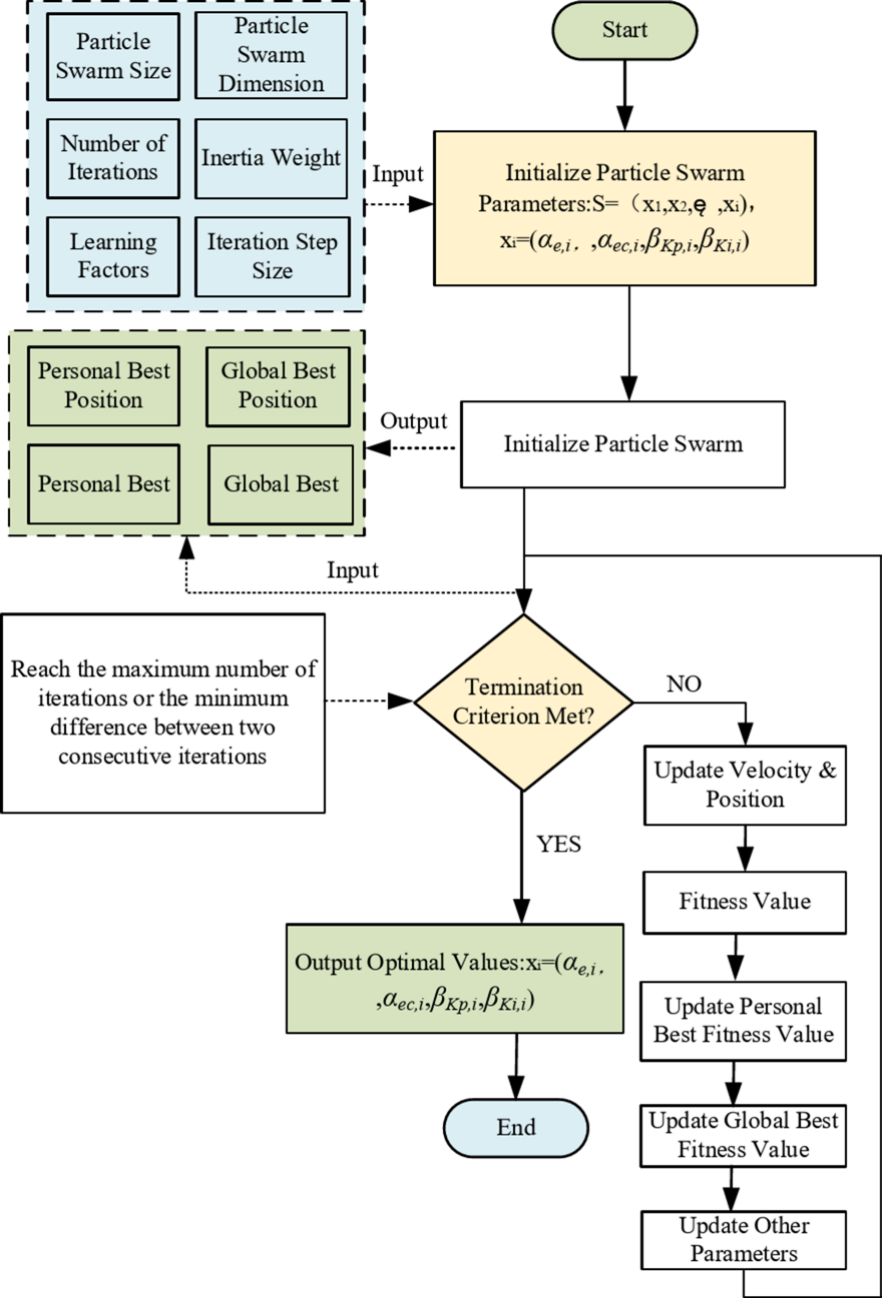
Particle swarm optimisation flow chart.

**Fig. 6:**
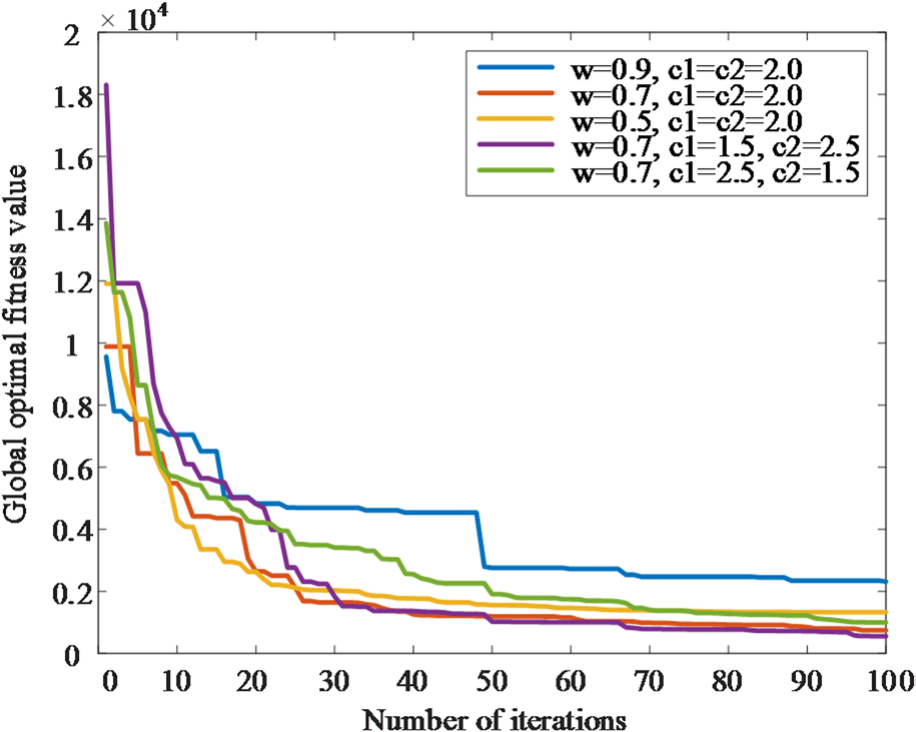
Convergence comparison with different PSO parameter settings.

Through iterative optimisation, the PSO algorithm produces an optimal parameter set [*α*_*e*_, *α*_*ec*_, *β*_*Kp*_, *β*_*Ki*_] (Fig. 5). To illustrate how these parameters regulate the fuzzy PI controller, Fig. 6 presents its principle block diagram with embedded PSO optimisation. Specifically, the quantisation factors *α*_*e*_ and *α*_*ec*_ are applied to the input membership functions, while the proportional and integral scaling factors *β*_*Kp*_ and *β*_*Ki*_ are incorporated into the defuzzification stage.

To further clarify the optimization process and parameter selection, additional convergence experiments were performed with varying PSO configurations, as summarized below.The main configurations used in the PSO convergence experiments are listed in Table 3.

**Table 3:** Main setup of PSO optimization experiments.

Symbol	Meaning	Value / Range
Np	Particle population size	20, 30, 50
Gmax	Iteration limit	50,100
w	Inertia weight	0.5–0.9
*c*_1_,*c*_2_	Cognitive & social coefficients	1.5–2.5
Boundary	Search range	[-100, 100]
Fitness function	Sum of squared parameters (minimization)	Time-weighted absolute error integral (ITAE)
Random seeds	Number of independent runs	10 per configuration

Each configuration was executed 10 times with different random seeds to evaluate the influence of stochasticity and to compute the mean, standard deviation, and final convergence fitness.

The convergence curves under different PSO parameter settings are shown in Fig. 6, where the horizontal axis represents the number of iterations and the vertical axis represents the global optimal fitness value.

As shown in Fig. 6, all tested settings exhibit a rapid decrease of the global-best fitness within the first 20–30 iterations, followed by a gradual stabilization. Larger inertia weights (e.g., *w*→0.9) slow down exploitation and may leave a higher terminal fitness, whereas too small w accelerates convergence but increases the risk of premature stagnation. Setting *c*_1_and *c*_2_in the range 1.5–2.5 yields stable descent without oscillatory behavior. Across 10 runs per configuration, the final fitness variability remained within ±(5–7)%, indicating good repeatability.

Based on the above trade-off between convergence speed and solution quality, this paper adopts *w* = 0.7, *c*_1_ = 1.5, *c*_2_ = 2.5, Np = 100, Gmax = 50, and the search range (*α*,*β*)∈[0,30]. This setting consistently reached near-stationary fitness around 60 iterations while achieving the lowest average terminal fitness among the tested candidates, thereby providing high-quality references for the outer-loop controller at modest computational cost.

**Fig. 7:**
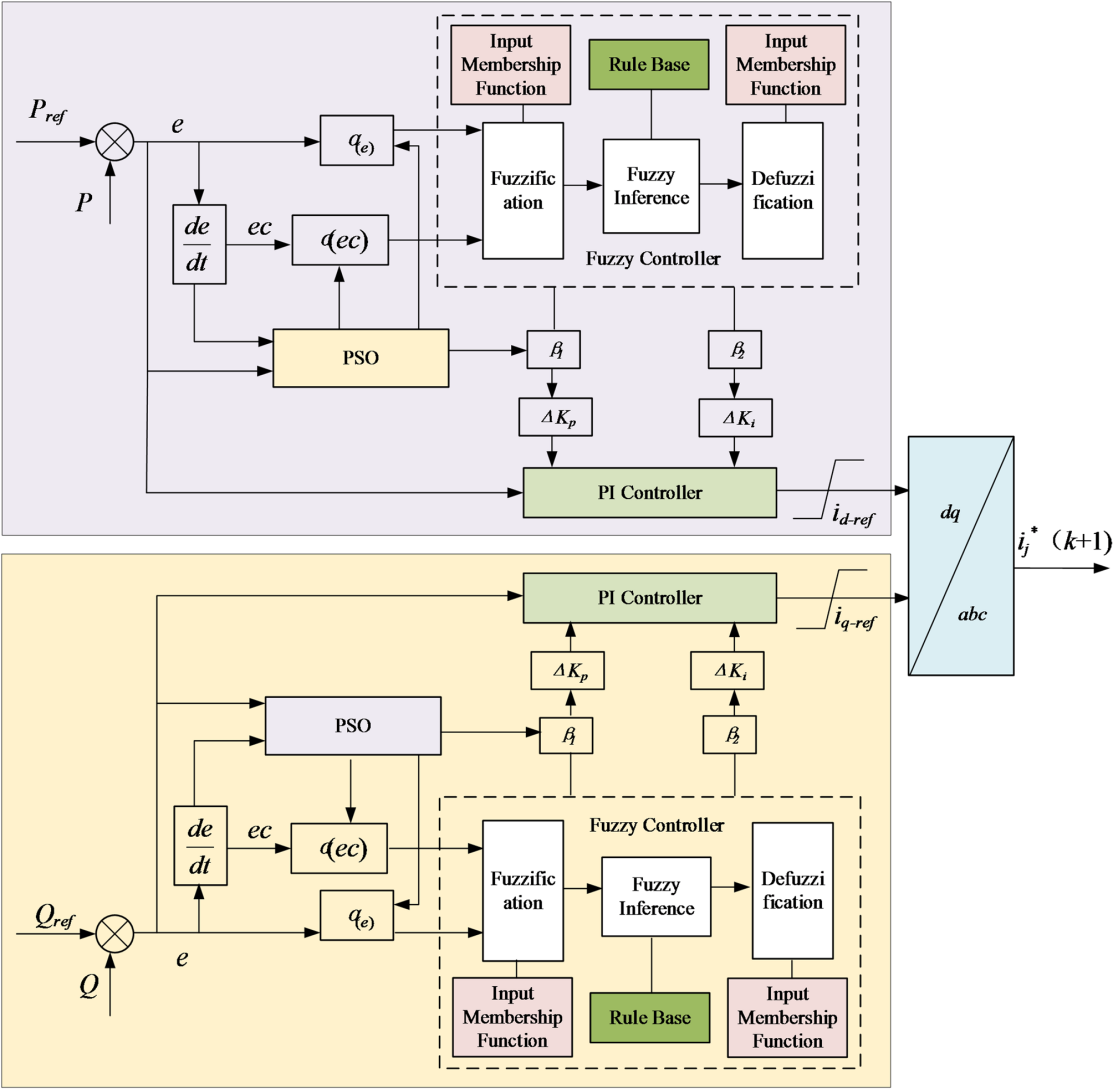
PSO-optimized fuzzy PI controller for outer-loop power control and dq-axis current reference generation.

Within the closed-loop control, the fuzzy reasoning mechanism continuously adjusts the PI parameters (*ΔK*_*p*_, *ΔK*_*i*_) according to the active/reactive power error (*e*) and its rate of change (*ec*). This process generates the d- and q-axis current references (*i*_*d-ref*_, *i*_*q-ref*_). As shown in Fig. 7, these references are converted back into the three-phase frame through the inverse *dq*–*abc* transformation, producing the predicted current references *i*_*j*_^∗^(*k* + 1). The one-step-ahead references are then supplied to the inner-loop Model Predictive Control (MPC), which executes current tracking, suppresses circulating currents, and balances capacitor voltages. This coordinated design enables effective synergy between the outer fuzzy PI controller and the inner MPC.

### Design of the inner-loop finite-state hierarchical MPC

**Fig. 8:**
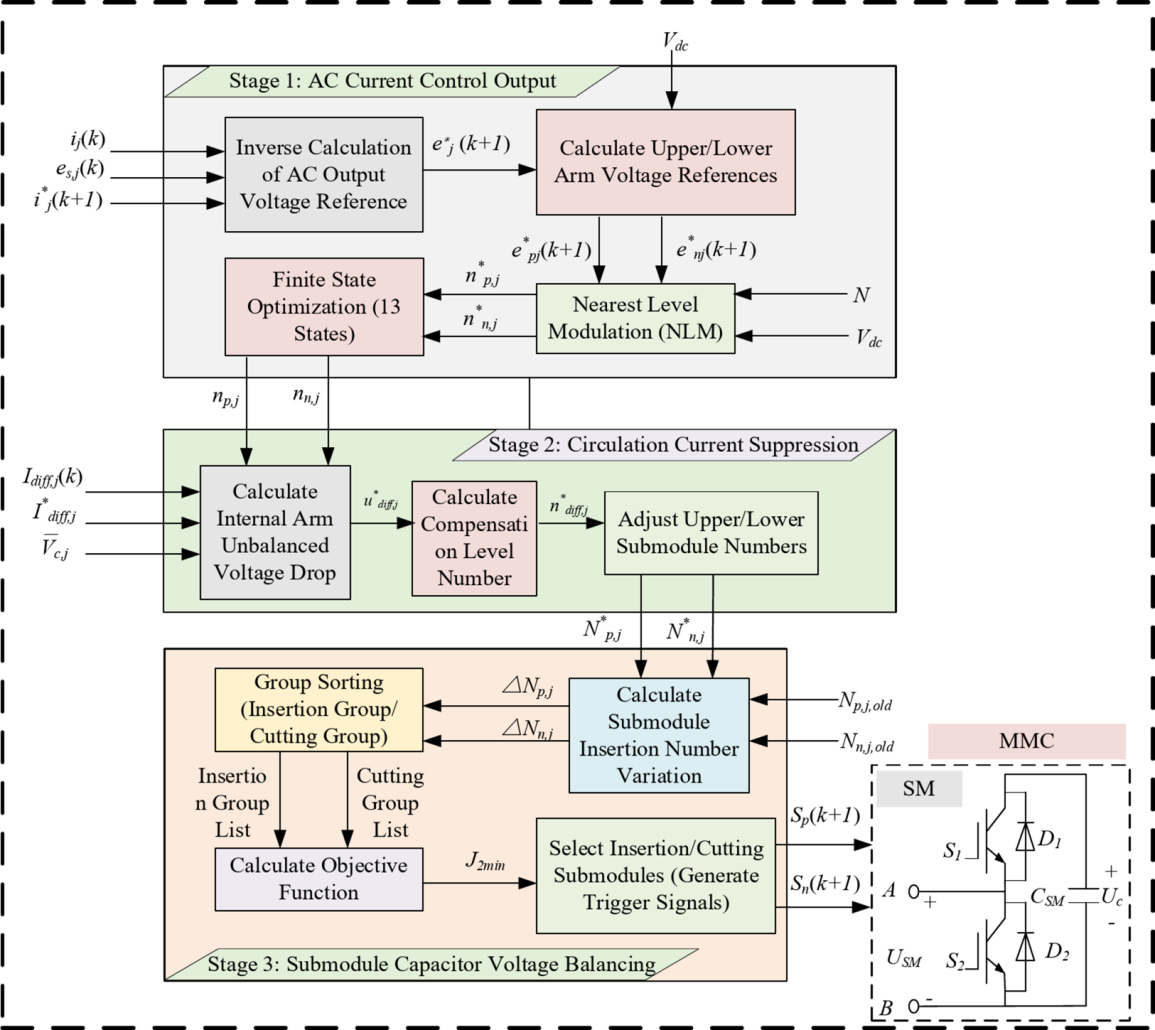
Block diagram of finite state hierarchical MPC control structure.

The inner loop adopts a finite-state hierarchical model predictive control (FSH-MPC) strategy. Its core idea is to decouple multiple objectives into a hierarchical structure consisting of three progressive layers: current tracking, circulating current suppression, and capacitor voltage balancing. Each layer performs local finite-state optimisation or correction under the reference and constraints provided by the preceding layer. In this way, the method avoids exhaustive evaluation of all (*N* + 1)^2^ states, thereby satisfying the real-time requirements of the sampling period. The overall control structure is shown in Fig. 8.

The control of the AC-side current constitutes the core of the inner loop. The reference currents are provided by the outer-loop power controller, while the inner loop ensures their fast tracking to achieve accurate power delivery. Based on the equivalent circuit of the MMC AC side, the dynamic current equation (Eq. 7) is discretised using a first-order forward difference, yielding the current prediction model as shown in Eq. (24):

24 $${i_j}(k+1)=\frac{1}{{R+\frac{{{L_{eq}}}}{{{T_s}}}}}\left[ {{u_j}(k+1) - {u_{s,j}}(k+1)+{i_j}(k)\frac{{{L_{eq}}}}{{{T_s}}}} \right]$$

where *L*_*eq*_=*L* + *L*_*0*_/2 represents the equivalent inductance.

Based on the current prediction model given in Eq. (24), the reference AC-side voltage *u*_*s, j*_^∗^(*k* + 1) can be inversely obtained as follows:

25 $$\begin{array}{*{20}{c}} {u_{{s,j}}^{ * }(k+1)={u_{s,j}}(k+1)+Ri_{j}^{ * }(k+1)+\frac{{{L_{eq}}}}{{{T_s}}}\left( {i_{j}^{ * }(k+1) - {i_j}(k)} \right)} \end{array}$$

Given the short sampling period *T*_*s*_, the approximation *u*_*s, j*_(*k* + 1) ≈ *u*_*s, j*_(*k*) is adopted. The approximation error is corrected in the next cycle through current feedback, preventing cumulative deviation.

Based on the topological relationship between the upper and lower arm voltages and the AC output voltage, the bridge-arm voltage references can be derived as:

26 $$\left\{ {\begin{array}{*{20}{c}} {u_{{p,j}}^{ * }(k+1)=\frac{{{V_{dc}}}}{2} - u_{j}^{ * }(k+1)} \\ {u_{{n,j}}^{ * }(k+1)=\frac{{{V_{dc}}}}{2}+u_{j}^{ * }(k+1)} \end{array}} \right.$$

Using the nearest-level approximation (NLA), the initial numbers of inserted submodules are obtained as:

27 $$\left\{ {\begin{array}{*{20}{c}} {n_{{p,j}}^{ * }=round\left( {u_{{p,j}}^{ * }(k+1)\frac{N}{{{V_{dc}}}}} \right)} \\ {n_{{n,j}}^{ * }=round\left( {u_{{n,j}}^{ * }(k+1)\frac{N}{{{V_{dc}}}}} \right)} \end{array}} \right.$$

where round denotes the rounding-to-nearest operator, *N* is the number of submodules per arm, and *n*_*p, j*_^∗^,*n*_*n, j*_^∗^ represent the initially estimated numbers of submodules in the upper and lower arms, respectively. However, because the rounding process inevitably reduces phase current tracking accuracy, and the true optimum is subject to model and parameter perturbations, the effective solution generally fluctuates slightly around these initial estimates.

To capture the optimal switching combination while maintaining current tracking precision, local enumeration is performed within the range [*N* − *δ*,*N* + *δ*]. This ensures that the AC-side voltage can realise the full 2*N* + 1 levels, thereby avoiding output degradation. In this work, *N* = 20 and *δ* = 2, which results in 13 valid switching combinations. The corresponding degrees of freedom are listed in Table 4.

**Table 4:** Thirteen switching state candidates with freedom degree (*N* = 20, δ= 2).

Serial number	Upper Arm Submodule Number	Lower Arm Submodule Number	Total Arm Submodule Number
1	$n_{{p,j}}^{ * }$	$n_{{n,j}}^{ * }$	20
2	$n_{{p,j}}^{ * }+1$	$n_{{n,j}}^{ * } - 1$	20
3	$n_{{p,j}}^{ * } - 1$	$n_{{n,j}}^{ * }+1$	20
4	$n_{{p,j}}^{ * }+2$	$n_{{n,j}}^{ * } - 2$	20
5	$n_{{p,j}}^{ * } - 2$	$n_{{n,j}}^{ * }+2$	20
6	$n_{{p,j}}^{ * }+1$	$n_{{n,j}}^{ * }$	21
7	$n_{{p,j}}^{ * }+2$	$n_{{n,j}}^{ * }$	22
8	$n_{{p,j}}^{ * }$	$n_{{n,j}}^{ * }+1$	21
9	$n_{{p,j}}^{ * }$	$n_{{n,j}}^{ * }+2$	22
10	$n_{{p,j}}^{ * } - 1$	$n_{{n,j}}^{ * }$	19
11	$n_{{p,j}}^{ * } - 2$	$n_{{n,j}}^{ * }$	18
12	$n_{{p,j}}^{ * }$	$n_{{n,j}}^{ * } - 1$	19
13	$n_{{p,j}}^{ * }$	$n_{{n,j}}^{ * } - 2$	18

The 13 candidate switching states are evaluated by substituting them into the current-tracking cost function:

28 $${J_1}=\left| {\begin{array}{*{20}{c}} {i_{j}^{ * }(k+1) - {i_j}(k+1)} \end{array}} \right|$$

The state yielding the minimum *J*_1_ is selected as the preliminary optimal numbers of inserted upper and lower submodules, *n*_*p, j*_ and *n*_*n, j*_.

Once the preliminary values of *n*_*p, j*_ and *n*_*n, j*_ are obtained from AC current control, suppression of the internal circulating current *i*_*diff, j*_ is required. Circulating currents arise from voltage imbalance between the bridge arms. To mitigate them without compromising the current-tracking objective, a voltage imbalance compensation strategy is applied within the hierarchical framework. According to Eq. (8), which describes the internal circulating-current dynamics, the overall voltage of each bridge arm can be further derived as shown in Eq. (29):

29 $${u_{1,j}}={u_{p,j}}+{u_{n,j}}={V_{dc}} - 2{L_f}\frac{{d{i_{diff,j}}}}{{dt}}={V_{dc}} - 2{u_{diff,j}}$$

where *u*_*diff, j*_ denotes the imbalance term. This term essentially represents the deviation between the DC-link voltage and the sum of the upper and lower arm voltages. Discretising Eq. (29) with a first-order forward difference yields the approximate imbalance voltage drop *u*_*diff, j*_^∗^:

30 $$u_{{diff,j}}^{ * }={L_0}\frac{{i_{{diff,j}}^{ * }(k+1) - {i_{diff,j}}(k)}}{{{T_s}}}$$

As indicated by Eq. (30), *u*_*diff, j*_^∗^ depends on the difference between the circulating current reference and its actual value. To compensate this imbalance, the number of inserted submodules must be adjusted. Based on the average capacitor voltage *V*_*c, avg*_, the required number of compensation levels is calculated as:

31 $$n_{{diff,j}}^{ * }=round\left( {\frac{{u_{{diff,j}}^{ * }}}{{{V_{c,avg}}}}} \right)$$

To avoid affecting the AC-side voltage, an equal number of compensation submodules is simultaneously adjusted in both arms. Finally, by incorporating the compensation levels, the corrected numbers of inserted submodules are obtained:

32 $$\left\{ {\begin{array}{*{20}{c}} {N_{{p,j}}^{ * }={n_{p,j}}+n_{{diff,j}}^{ * }} \\ {N_{{n,j}}^{ * }={n_{n,j}}+n_{{diff,j}}^{ * }} \end{array}} \right.$$

where *N*_*p, j*_^∗^ and *N*_*n, j*_^∗^ denote the final numbers of inserted submodules in the upper and lower arms, respectively.

To ensure capacitor voltage balancing while maintaining real-time control, a voltage-prediction-based group sorting strategy is adopted. The predicted submodule capacitor voltage is calculated as:

33 $${u_{c,m,j,i}}(k+1)={u_{c,m,j,i}}(k)+\frac{{{i_{m,j}}(k){T_s}}}{C}$$

where *u*_*c, m,j, i*_ denotes the capacitor voltage of the *i*-th submodule in the upper (*m* = *p*) or lower (*m* = *n*) arm of phase *j*, and *i*_*m, j*_ is the corresponding arm current.

Each submodule voltage is predicted for the next sampling period. If a submodule is bypassed, its voltage is assumed constant; if inserted, the change is computed by Eq. (33). A capacitor-voltage balancing cost function is then constructed using the predicted value, with its minimum taken as the sorting index:

34 $${J_{2,m,j,i}}=\frac{{{i_{m,j}}(k){T_s}}}{C}\left[ {{u_{c,m,j,i}}(k+1) - \frac{{{V_{dc}}}}{N}} \right]$$

where *u*_c, m,j, i_(*k* + 1) denotes the predicted capacitor voltage of the *i*-th submodule in the *m*-th arm of phase *j*, and *i*_m, j_ represents the corresponding arm current. Minimizing *J*_2,*m*,*j*,*i*_ allows the controller to determine which submodules should be inserted or bypassed, thus achieving capacitor-voltage equalization among all submodules.

During steady-state operation, the change in the number of inserted submodules per period is typically − 1, 0, or + 1. To reduce computation, a predictive group-sorting strategy is adopted: each arm’s submodules are divided into two groups: inserted and bypassed, each group is sorted independently.

When Δ*N* > 0 (additional submodules need to be inserted), the Δ*N* submodules corresponding to the smallest values of the objective function *J*_2,*m*,*j*,*i*_ are selected from the bypassed group for insertion.

When Δ*N* < 0 (submodules need to be bypassed), the Δ*N* submodules with the smallest *J*_2,*m*,*j*,*i*_ values are selected from the inserted group for bypassing. Other submodules retain their previous states.

35 $$\left\{ {\begin{array}{*{20}{c}} {\Delta {N_{\mathrm{p},j}}=N_{{\mathrm{p},j,\mathrm{n}\mathrm{e}\mathrm{w}}}^{ * } - N_{{\mathrm{p},j,\mathrm{o}\mathrm{l}\mathrm{d}}}^{ * }} \\ {\Delta {N_{\mathrm{n},j}}=N_{{\mathrm{n},j,\mathrm{n}\mathrm{e}\mathrm{w}}}^{ * } - N_{{\mathrm{n},j,\mathrm{o}\mathrm{l}\mathrm{d}}}^{ * }} \end{array}} \right.$$

where: *N*^∗^_*p*,*j, new*_ and *N*^∗^_*n, j,new*_ denote the numbers of inserted submodules in the upper and lower arms at time *k* + 1, respectively; *N*^∗^_*p, j,old*_ and *N*^∗^_*n, j,old*_ denote those at time *k*.

During each control cycle, capacitor voltages are grouped and sorted for every arm. When Δ*N* > 0, Δ*N* submodules are selected from the bypassed group; when ΔN < 0, ΔN are removed from the inserted group, while the rest remain unchanged. The process is illustrated schematically below, where *S*_*s, j,k*_ represents the submodule switching signal, *Q* the objective function value, and *X* an intermediate sorting variable.

**Fig. 9:**
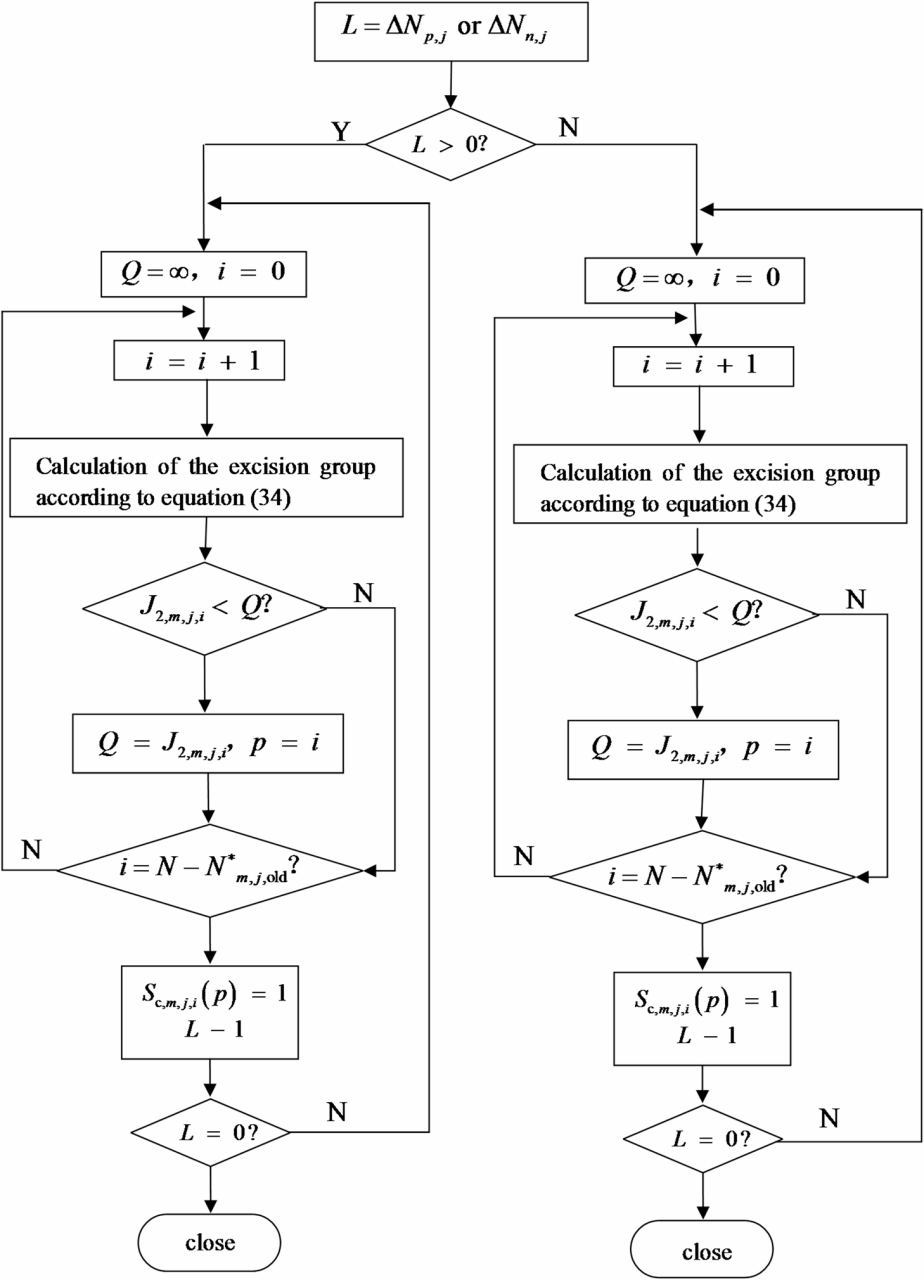
Flowchart of predictive group-sorting for submodule capacitor-voltage balancing.

Based on (34)–(35), the single-cycle implementation of the predictive group-sorting procedure is summarized in Fig. 9. Starting from *L* = Δ*N*_p, j_ or Δ*N*_n, j_, the algorithm evaluates *J*_*2,j, m,i*_ for the “inserted” and “bypassed” groups according to (34), selects ΔN submodules with the smallest cost, and updates the switching state *S*_*s, j,k*_ accordingly.

Overall, the proposed inner-loop controller first determines the preliminary number of inserted submodules through inverse prediction of the AC-side voltage, and then selects the optimal one among 13 effective candidate states to achieve accurate current tracking. Circulating currents are suppressed by compensating voltage imbalance, which eliminates the need for weight factors while still maintaining the full 2*N* + 1 output voltage levels. Meanwhile, for capacitor voltage balancing, a prediction-based group sorting strategy is adopted: submodules are divided into inserted and bypassed groups and sorted by cost function within each group, and only the submodules that require switching are adjusted while the others remain unchanged. This approach avoids exhaustive searching of all submodules, reduces computational burden, balances capacitor voltages, and minimizes unnecessary switching losses.

In contrast, conventional finite-state MPC must evaluate (*N* + 1)^2^ states. The proposed method requires only 31 operations per control cycle (13 optimizations, 13 predictions, 2 inverse derivations, and 3 conduction updates). Its complexity is independent of *N*, achieving about 93% reduction when *N* = 20, with the advantage becoming more pronounced as N increases.

## Simulation results and performance analysis

To validate the effectiveness of the proposed MMC dual-loop cooperative control strategy, which integrates a PSO-optimized fuzzy PI controller in the outer loop with an FCS-MPC scheme in the inner loop, a simulation model of the MMC system was developed in MATLAB/Simulink. The main system parameters are summarized in Table 5, where the fuzzy PI controller parameters were tuned offline using PSO, and the corresponding optimization settings are also listed.

**Table 5:** Protection operation time for different fault locations and fault resistances.

Parameter	数值
Submodules per arm, N	20
Submodule capacitance, C/mF	9
DC-link voltage, U_dc_/kV	60
AC system voltage, e_g_/kV	22
AC frequency, f /Hz	50
Arm inductance, L_0_/mH	20
AC inductance, L/mH	10
AC resistance, R/Ω	0.1
Sampling period, T/µs	50

The system is initialized at steady state with reactive power reference set to Q^*^ = 150 kvar and active power reference set to P^*^ = 300 kW. To evaluate the transient performance of the improved control method, the reactive power is stepped to Q^*^ = 80 kvar at *t* = 0.05 s, followed by an active power step to P^*^ = 1000 kW at *t* = 0.1 s.

**Fig. 10:**
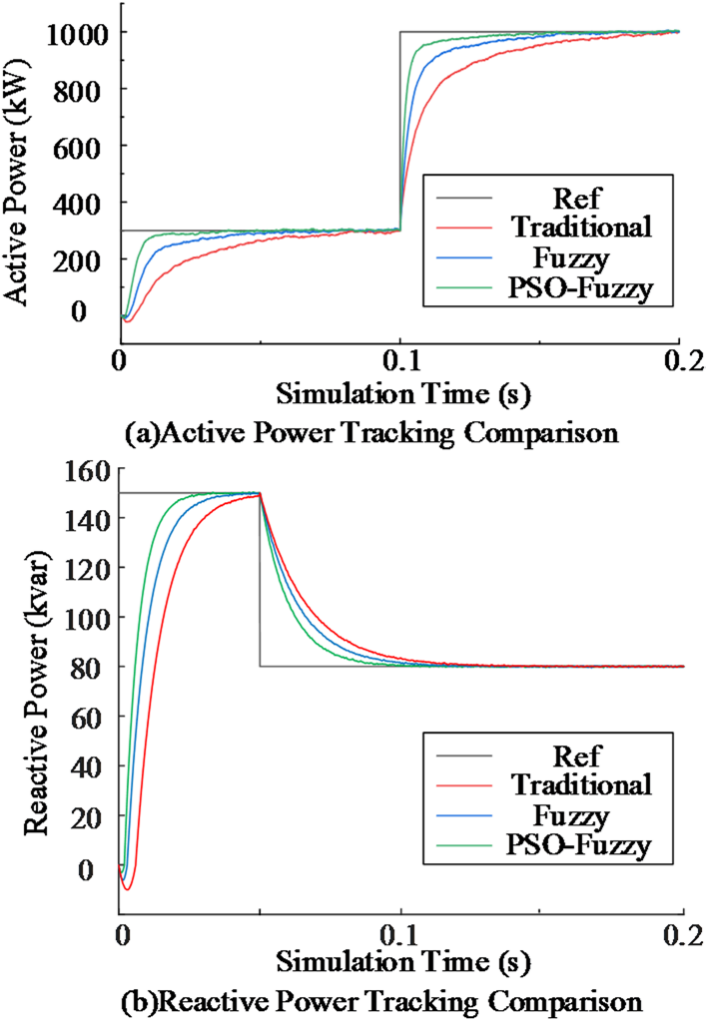
Comparison of power tracking under three control strategies.

To further assess the impact of different outer-loop strategies on dynamic performance, three approaches—conventional PI, fuzzy PI, and PSO-optimized fuzzy PI—are compared under the same step conditions. The corresponding active and reactive power tracking results are presented in Fig. 10.

As shown in Fig. 10(a), all three control strategies achieve active power tracking, but noticeable differences appear in transient response speed and steady-state accuracy. To facilitate quantitative comparison, the settling time, overshoot, and steady-state error are summarized in Table 6.

**Table 6:** Active power tracking performance comparison of three control methods.

Controller	Settling time (ms)	Overshoot (%)	Steady-state error (%)
Traditional PI	48.09	3.30	5.74
Fuzzy PI	24.99	2.03	3.22
PSO-Fuzzy PI	8.04	0.85	2.25

As indicated in Table 6, the PSO-fuzzy PI reduces settling time by 67.9%, overshoot by 58.1%, and steady-state error by 30.1% compared with the fuzzy PI. Relative to the conventional PI, the reductions are 83.3%, 74.2%, and 60.8%, respectively. The reactive power responses in Fig. 10(b) exhibit a similar trend, with the PSO-fuzzy PI consistently delivering superior tracking performance. These results confirm that the proposed strategy offers significant advantages in both dynamic rapidity and steady-state precision.

**Fig. 11:**
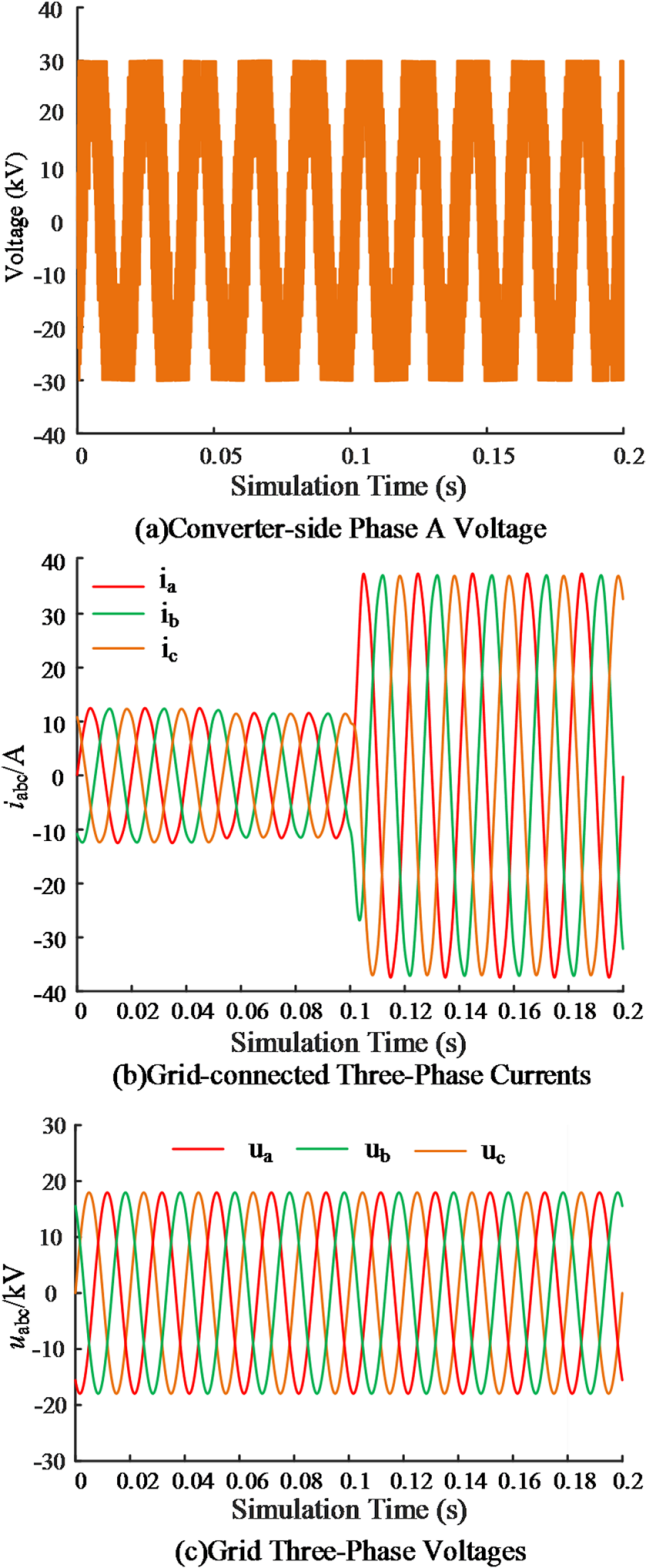
Comparison of simulation results of AC side output.

To validate the proposed control strategy in terms of AC-side output characteristics and its influence on grid-connected waveforms, simulations were performed on the converter output. The results are shown in Fig. 11.

Figure 11 (a) presents the phase-a converter voltage, which exhibits a distinct stepped waveform characteristic of modular multilevel operation. The number of inserted submodules per bridge arm varies rather than being fixed at *N*, thereby enabling the maximum 2*N* + 1 output levels. Figure 11 (b) shows the grid-connected three-phase current, which is nearly sinusoidal with high tracking accuracy. Step disturbances are rapidly suppressed without noticeable oscillations. Figure 11 (c) illustrates that the grid voltage maintains a sinusoidal profile without significant distortion or additional perturbations.

**Fig. 12:**
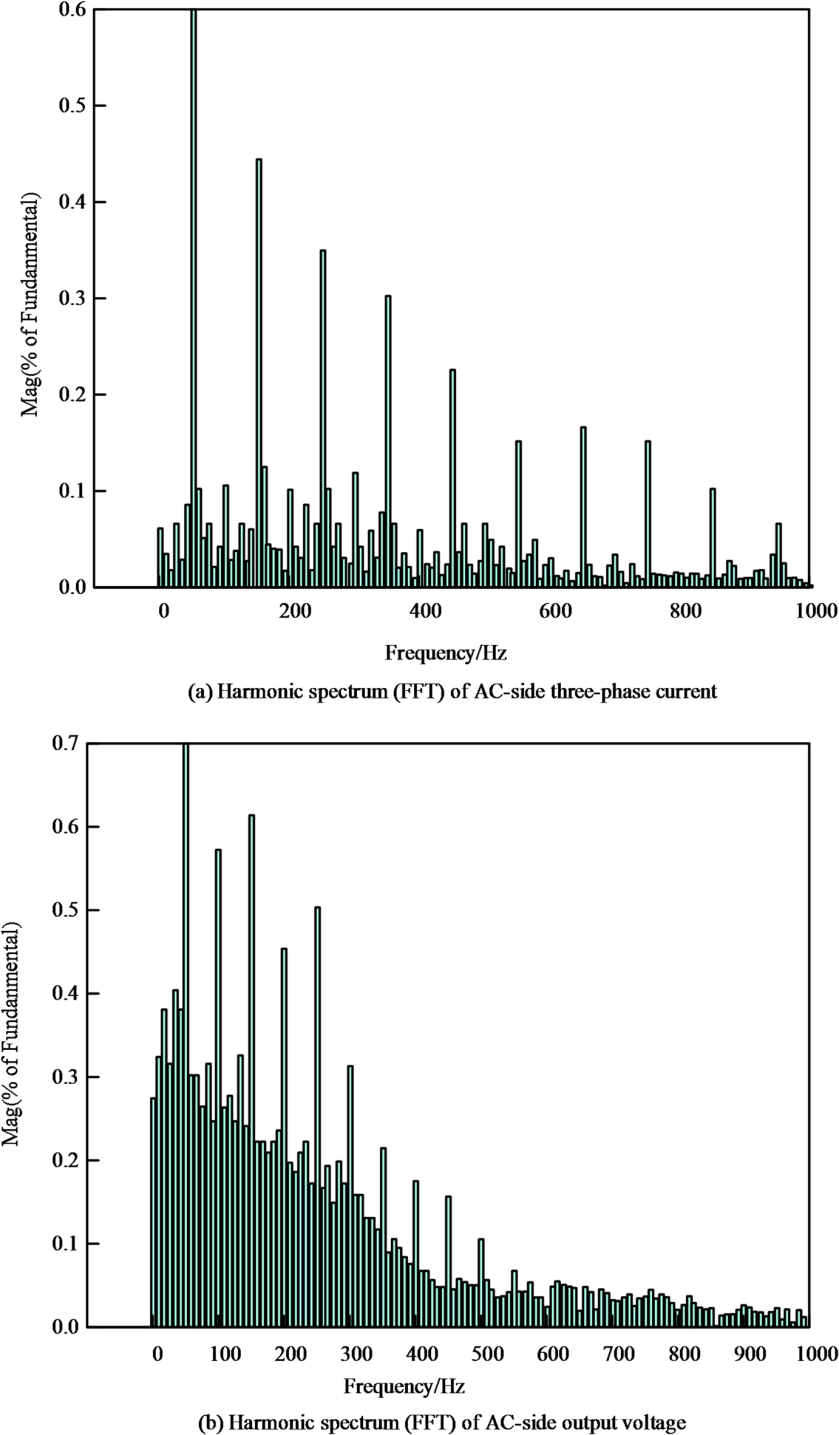
Comparison of simulation results of AC side output.

To further evaluate waveform quality, the harmonic spectra of the AC-side current and voltage are analyzed using FFT, as shown in Fig. 12. From Fig. 12(a), the calculated total harmonic distortion (THD) of the three-phase current is 1.27%, indicating excellent current quality and strong harmonic suppression achieved by the proposed cooperative control strategy. From Fig. 12(b), the THD of the AC-side output voltage is 2.13%, confirming that the hierarchical MPC in the inner loop effectively ensures high-quality voltage output and low-distortion performance under steady-state conditions.

**Fig. 13:**
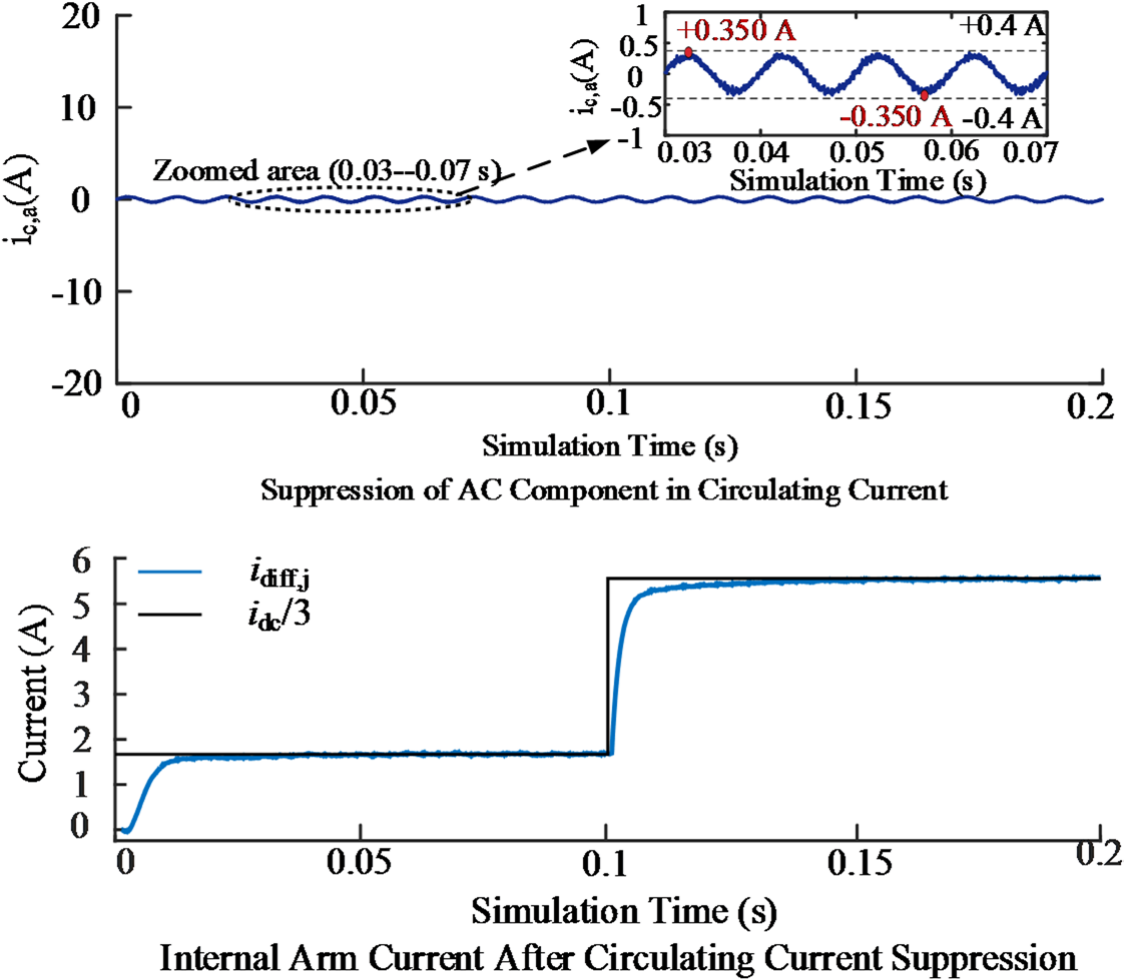
Simulation results of Phase A circulating current suppression and internal unbalanced current under the proposed method.

The circulating current in MMC bridge arms increases device losses and compromises the stability of the DC-side voltage. To evaluate the suppression capability of the proposed inner-loop control, the A-phase of the MMC was analyzed, focusing on the circulating current and the internal unbalanced current. The simulation results are shown in Fig. 13.

Figure 13 (a) indicates that the peak AC circulating current is constrained within ± 0.35 A, with the low-frequency component significantly attenuated. Figure 13 (b) shows that the internal unbalanced current maintains a stable DC component around I_dc_/3, with values of approximately 1.67 A under steady state and 5.56 A after the step change. The maximum fluctuation is limited within 5%, while harmonic components are effectively suppressed. These results confirm the effectiveness of the proposed circulating current suppression strategy in the inner-loop control.

**Fig. 14:**
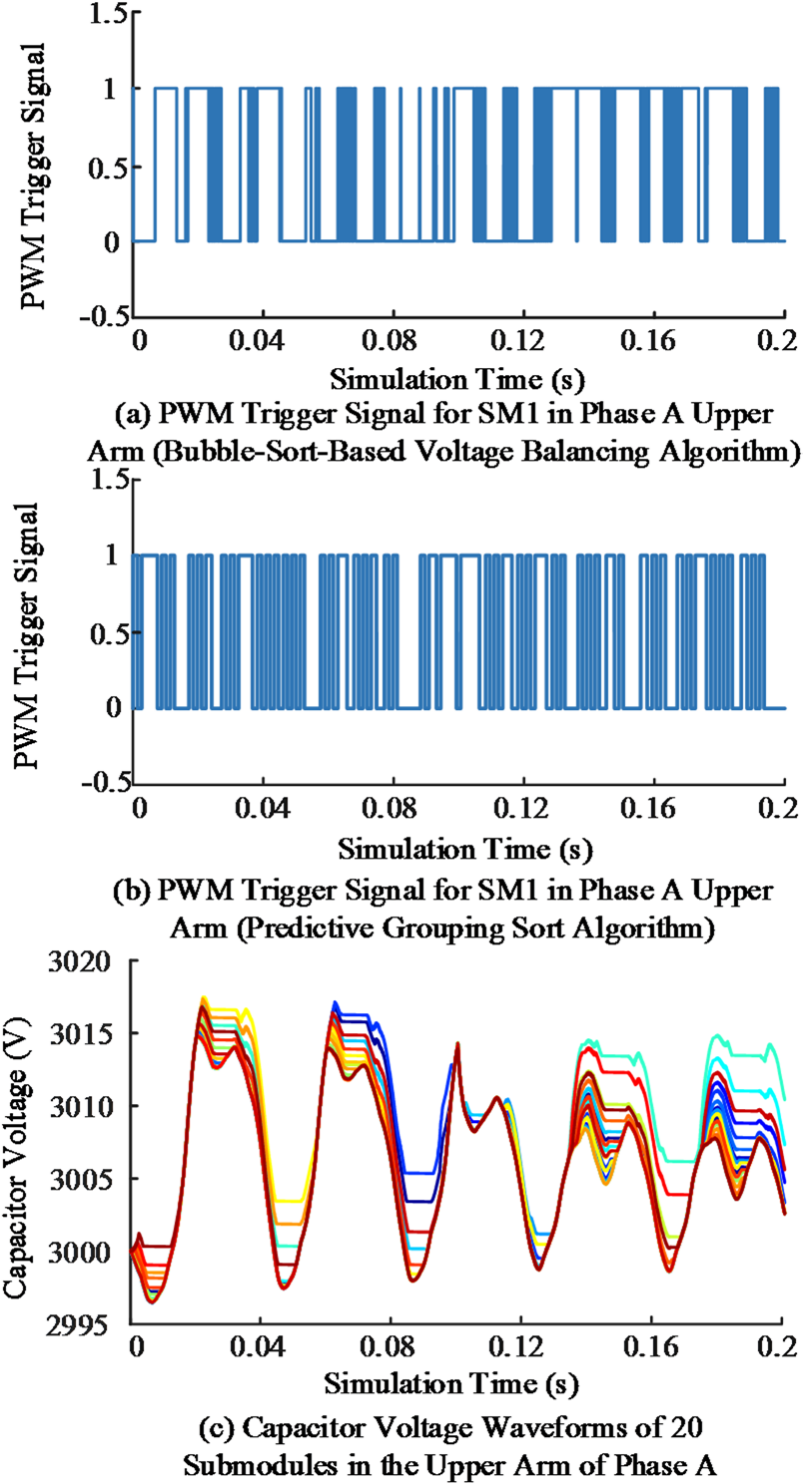
Simulation results of PWM pulse characteristics and capacitor voltage equalisation under different sequencing strategies.

To evaluate the influence of different submodule trigger sequencing strategies on switching frequency and capacitor voltage balancing, the PWM characteristics and capacitor voltage distribution of SM1 in the A-phase upper arm were compared under the Bubble-Sort-Based Voltage Balancing Algorithm and the Predictive Group Sequencing Algorithm. The simulation results are shown in Fig. 14.

Figure 14 (a) shows that the Bubble-Sort-Based method yields an average switching frequency of about 980 Hz. In contrast, Fig. 14 (b) indicates that the Predictive Group Sequencing method reduces the average frequency to approximately 530 Hz, representing a 46% decrease. Figure 14 (c) further demonstrates that the capacitor voltages of the 20 submodules in the A-phase upper arm are maintained within 2995–3020 V, with the maximum deviation limited to ± 0.67%. These results confirm that the proposed capacitor voltage balancing strategy achieves stable capacitor voltages while significantly lowering the switching frequency.

## Conclusion

This paper proposed and validated a cooperative control strategy that integrates a PSO-optimized fuzzy PI outer loop with a hierarchical FCS-MPC inner loop. In the outer loop, the PSO algorithm performed an offline global optimization of the fuzzy PI scaling and gain parameters, enabling adaptive tuning and generating high-quality current references for accurate power tracking. The hierarchical FCS-MPC in the inner loop reduced the computational burden of prediction and optimization while maintaining accurate current tracking. Circulating-current suppression was achieved through a compensation-level mechanism derived from inter-arm voltage imbalance, and the predictive grouping–sorting scheme further reduced switching frequency and improved capacitor-voltage balancing. Through the coordinated interaction between the two loops, the proposed strategy achieves faster dynamics, improved steady-state accuracy, and reduced reliance on manual parameter tuning, showing clear advantages over conventional methods.

Since the PSO optimization is carried out in an offline manner, the obtained optimal parameters enable the controller to maintain stable and accurate performance under nominal and small-disturbance conditions; however, its adaptability under sustained or large-scale operating variations still has room for further enhancement. Future work will involve hardware-in-the-loop validation on the TI TMS320F28379D (DSP) and Xilinx Artix-7 (FPGA) platforms to evaluate real-time performance in terms of timing coordination and delay compensation, and to further explore the extension of the proposed cooperative framework to multi-terminal MMC-HVDC systems and renewable-energy integration, thereby promoting its practical deployment in engineering applications.

## Data Availability

The datasets used and/or analysed during the current study are available from the corresponding author upon reasonable request.
